# Supplementary material for: A human endothelial cell-based recycling assay for screening of FcRn targeted molecules
Source: Nat Commun. 2018 Feb 12;9:621. doi: 10.1038/s41467-018-03061-x (PMC5809500; doi:10.1038/s41467-018-03061-x)
Supplement: Supplementary file 1 — Supplementary Information [file 41467_2018_3061_MOESM1_ESM.pdf]

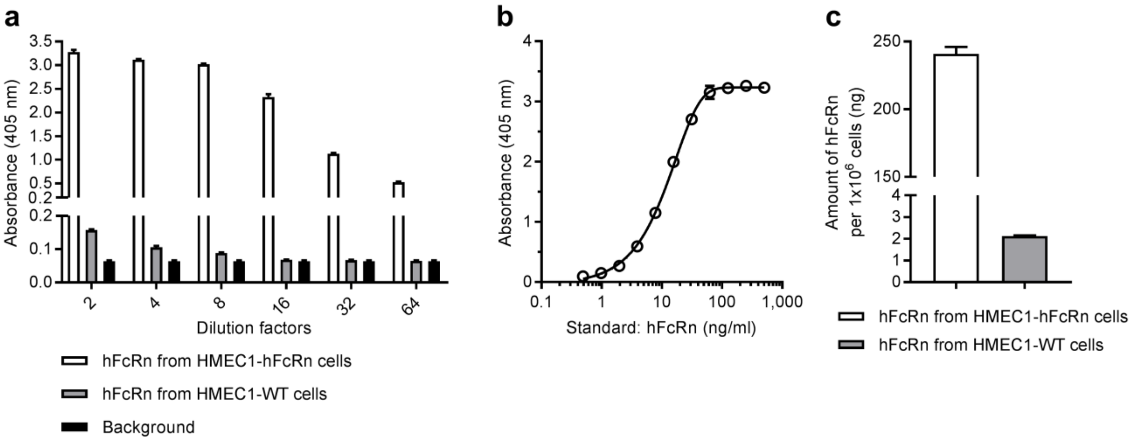

Supplementary Figure 1. Quantification of the level of hFcRn expression in HMEC1 cell lines. (**a**) ELISA measurements of the level of functional hFcRn expression in extracted membrane protein fractions isolated from the HMEC1-hFcRn and WT HMEC1 cell lines. The relative expression levels of the hFcRn HC were quantified in ELISA by capturing of the extracted receptor on hIgG1-YTE/KF coated in wells followed by detection using a biotinylated anti-hFcRn monoclonal antibody (ADM31). (**b**) ELISA measurements of titrated amounts (1,000.0-0.5 ng/ml) of recombinant soluble hFcRn used as a standard for quantification of hFcRn present in the extracted membrane protein fractions. (**c**) The amounts of hFcRn detected in the extracted fractions from 1.0×10^6 HMEC1-hFcRn or WT HMEC1 cells. Data are mean ± s.d. of one experiment performed in duplicates.

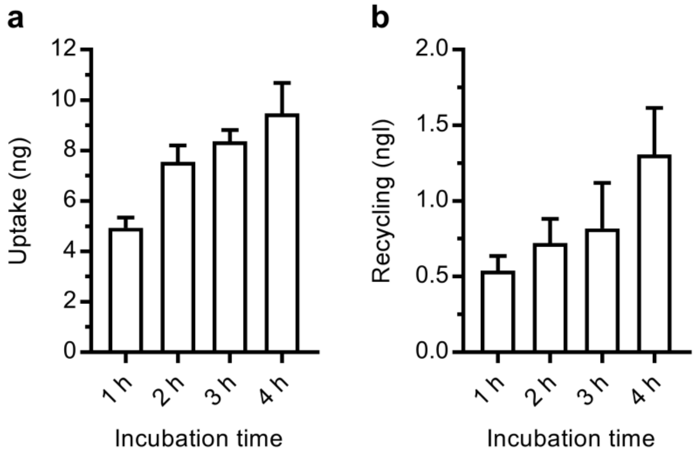

Supplementary Figure 2. Uptake and recycling dynamics of WT hIgG1. (**a**) Uptake of WT hIgG1 at pH 7.4 when 400 nM was added to the cells followed by incubation for 1 h, 2 h, 3 h and 4 h, washing and lysis of the cells. (**b**) Recycling of WT hIgG1 at pH 7.4 when 400 nM was added to the cells and incubated for 4 h followed by extensive washing and additional incubation for 1 h, 2 h, 3 h and 4 h before samples were collected. The amounts of WT hIgG1 in the samples were quantified by ELISA, and obtained data are shown as mean ± s.d. of two independent experiments performed in triplicates.

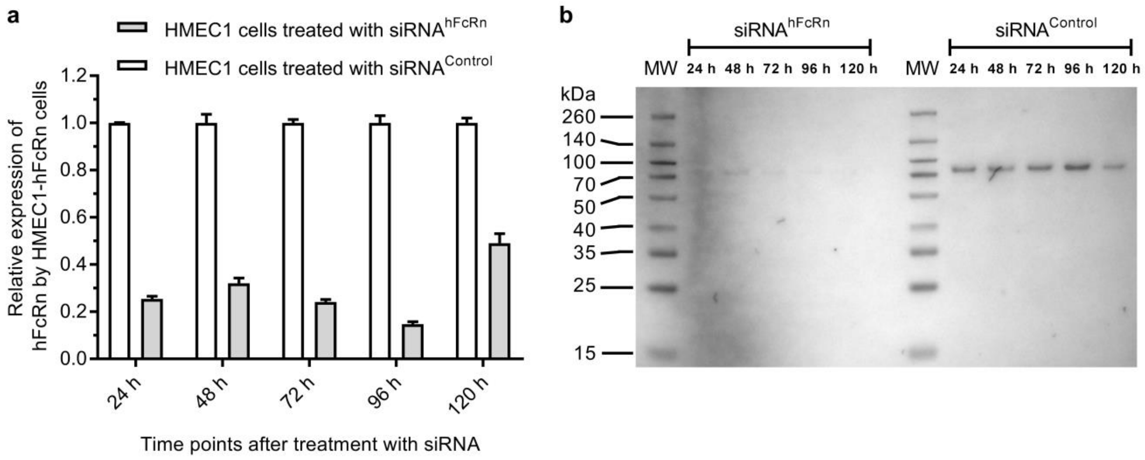

Supplementary Figure 3. **siRNA based downregulation of the hFcRn HC. (a)** HMEC1-FcRn cells were transfected with a mixture of control siRNA or siRNA targeting the gene of hFcRn HC. Cells were harvested after 24, 48, 72, 96 and 120 h post transfection followed by extraction of membrane protein fractions. The relative expression levels of the hFcRn HC were quantified in ELISA by capturing of the extracted receptor on hIgG1-YTE/KF coated in wells followed by detection using a biotinylated anti-hFcRn monoclonal antibody (ADM31). Obtained data are shown as mean ± s.d. of two experiments performed in duplicates. (**b**) A representative Western blot showing the expression level of hFcRn post treatment of HMEC1-FcRn cells with a mixture of control siRNA or siRNA targeting the FcRn HC at different time points (24, 48, 72, 96 and 120 h).

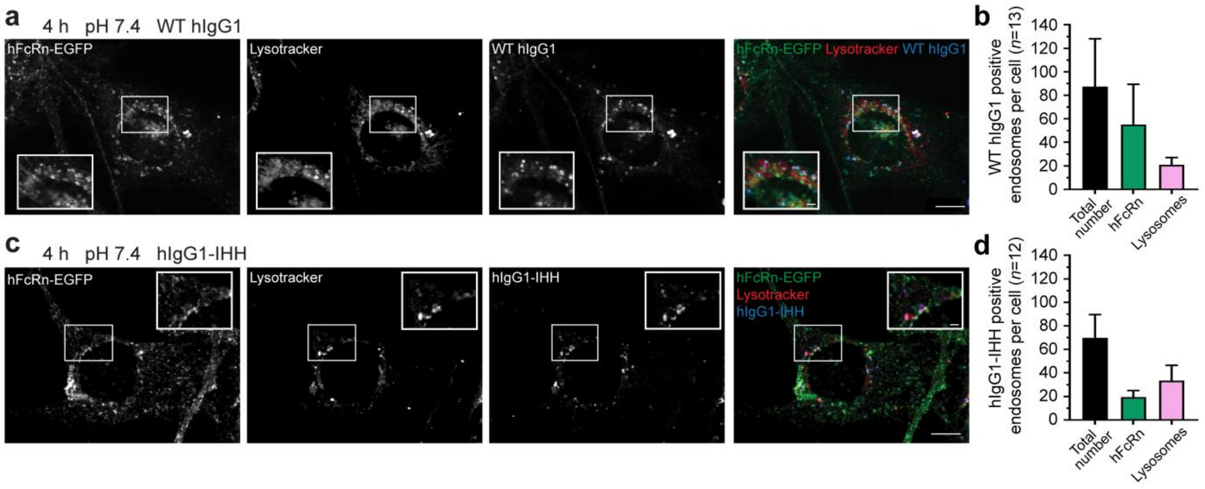

Supplemental Figure 4. Visualization of IgG uptake by HMEC1-hFcRn cells at neutral pH. HMEC1-hFcRn cells were seeded in 8-well Nunc™ Lab-Tek™ chambered Coverglass imaging dishes, and live cell imaging was performed the day after. (**a**) Cells were washed 3 times with HBSS pH 7.4 and incubated with 400 nM of Alexa-labelled WT hIgG1 diluted in HBSS pH 7.4 for 4 h at 37˚C. Cells were incubated with Lysotracker DND-99 for 30 min, before cells were washed with ice cold HBSS pH 7.4 and images were taken. (**b**) WT hIgG1 co-localization within FcRn positive endosomes or lysosomes after 4 h incubation (n = 13 cells). (**c**) Cells were washed 3 times with HBSS pH 7.4 and incubated with 400 nM of Alexa-labelled hIgG1-IHH diluted in HBSS pH 7.4 for 4 h at 37˚C. Cells were incubated with Lysotracker DND-99 for 30 min before cells were washed with ice cold HBSS pH 7.4 and images were taken. (**d**) hIgG1-IHH co-localization within FcRn positive endosomes or lysosomes after 4 h incubation (n = 12 cells). Representative confocal images of each condition are shown. Large scale bar - 10 µm, small scale bar - 2 µm. Co-localization analyses were performed by using Imaris spot co-localization. Data are shown as mean ± s.d. from two independent experiments were n = (**b**) 13 and (**d**) 12 cells.

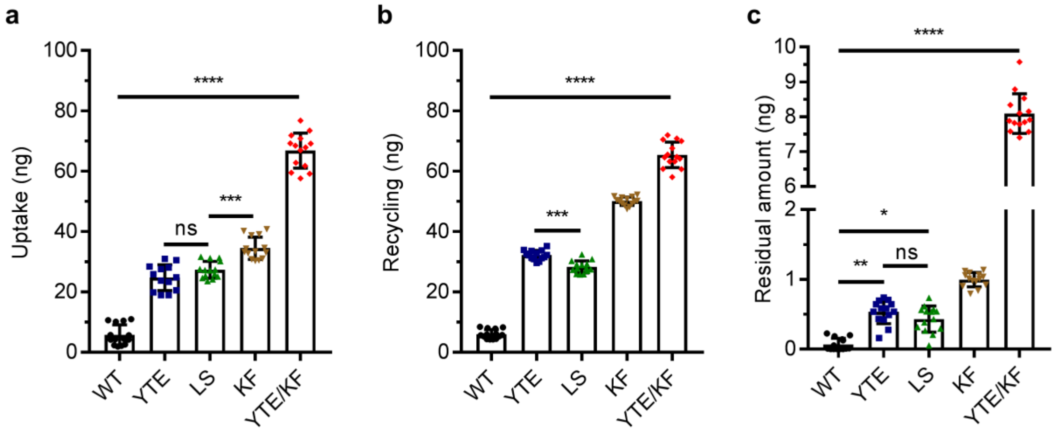

Supplementary Figure 5. Recycling of Fc-engineered IgG1 variants overnight. (**a**) Uptake of WT and Fc-engineered hIgG1 variants at pH 7.4. Portions of 400 nM of each variant were added to the cells followed by 4 hours incubation, washing and lysis of the cells. (**b**) 400 nM of WT and the Fc-engineered hIgG1 variants were added to cells and incubated for 4 hours followed by removal of medium and extensive washing. Warm medium was provided and samples were analysed after overnight incubation. (**c**) The same procedure as in (**b**) followed by lysis of the cells. The amounts of antibodies in all samples were quantified by ELISA. Obtained data are shown as mean ± s.d. of triplicates from 1 experiment. ns > 0.05, *p < 0.05, **p < 0.01, ***p < 0.001 and ****p < 0.0001, by one-way ANOVA (Tukey's multiple comparison test).

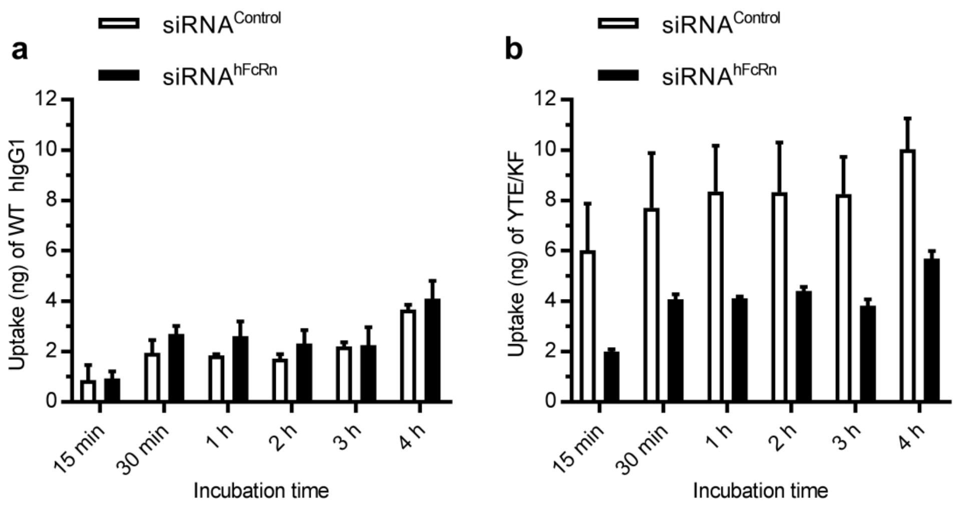

Supplementary Figure 6. Uptake dynamics of WT hIgG1 and YTE/KF. Uptake of (**a**) WT and (**b**) YTE/KF at pH 7.4 when 400 nM of each was added to HMEC-1-hFcRn cells treated with a mixture of control siRNA or siRNA targeting the hFcRn HC followed by incubation for 15 min, 30 min 1 h, 2 h, 3 h and 4 h. After washing, the cells were lysed and added to ELISA for quantification of the amounts of hIgG1 variants. Obtained data are shown as mean ± s.d. of one representative experiment performed in duplicates out of three independent experiments.

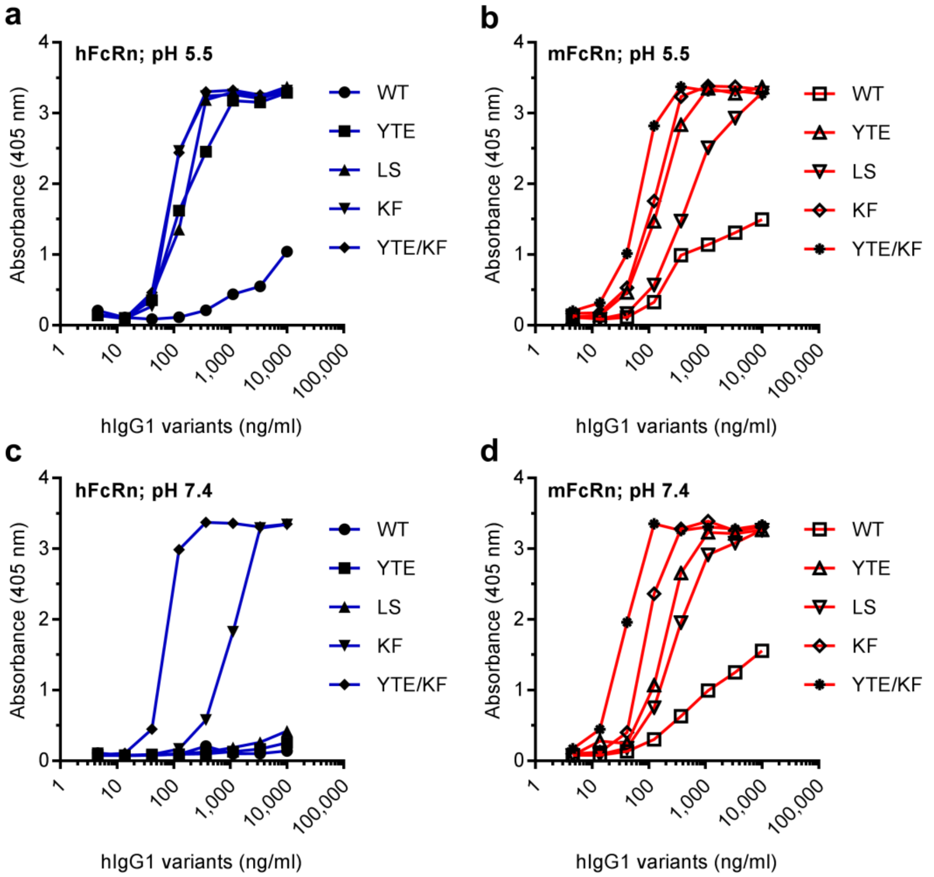

Supplementary Figure 7. Binding of hIgG1 variants to the mouse and human forms of FcRn. ELISA binding of titrated amounts (10,000.0-4.6 ng/ml) of WT hIgG1 and the Fc-engineered variants to (**a)** hFcRn and (**b**) mFcRn at pH 5.5 and (**c** and **d**) pH 7.4. Data represent OD values of one representative experiment run out of three.

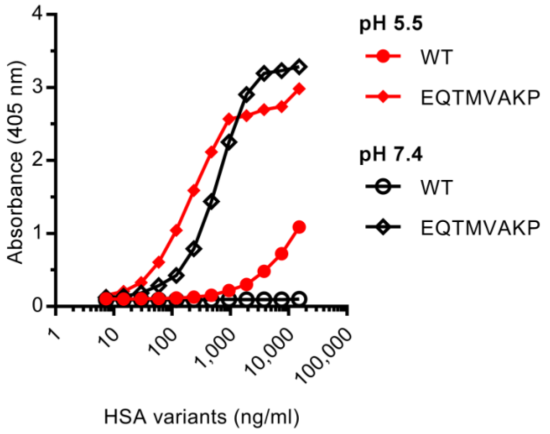

Supplementary Figure 8. Engineered HSA variant that binds strongly to hFcRn at pH 5.5 and pH 7.4. ELISA binding of titrated amounts (15,000-7.3 ng/ml) of WT HSA and EQTMVAKP to hFcRn at pH 5.5 and pH 7.4. Data are mean ± s.d. of one representative experiment out of three.

Supplementary Table 1. Analytical FcRn affinity chromatography analysis.

hIgG1 variants	Retention time (min)			pH value		
	start of peak	top of peak	end of peak	start of peak	top of peak	end of peak
WT	37.8	40.0	42.1	6.8	7.0	7.2
YTE	46.3	48.1	50.2	7.5	7.7	7.8
LS	47.2	49.8	53.2	7.6	7.8	7.9
KF	51.8	56.7	61.4	7.78	8.0	8.2
YTE/KF	72.6	77.6	83.0	8.5	8.6	8.7
HSA variants						
WT	38.1	43.1	47.0	6.0	6,4	6.7
K573P	44.3	48.7	51.0	6.5	6.9	7.3

Supplementary Table 2. Sequences of siRNAs targeting the HC of hFcRn.

siRNAs	Sequences
sc-45632A sense	5-GGAUCAAGGAGAAGCUCUUTT-3
sc-45632A antisense	5-AAGAGCUUCUCCUUGAUCCTT-3
sc-45632B sense	5-CUCACCUUCCUGCUAUUCUTT-3
sc-45632B antisense	5-AGAAUAGCAGGAAGGUGAGTT-3
sc-45632C sense	5-CGUCGUCACUAACAGUCAATT-3
sc-45632C antisense	5-UUGACUGUUAGUGACGACGTT-3
